# Prevalence and Correlates of Sleep Disorders Among Pediatric Inpatients in a Tertiary Pediatric Hospital

**DOI:** 10.7759/cureus.34871

**Published:** 2023-02-11

**Authors:** Sulhi Alfakeh, Rahaf L Mandili, Rajwa N Aljabri, Shaimaa H Salaam, Renad O Hamad, Hussam A Alhazmi, Maan A Samkari, Raghad S Alahmadi, Shouq Z Fatani, Ahmed K Bamaga, Abdullah M Khayat

**Affiliations:** 1 Department of Psychiatry, King Abdulaziz University Hospital, Jeddah, SAU; 2 General Practice, King Abdulaziz University Faculty of Medicine, Jeddah, SAU; 3 Medical School, King Abdulaziz University Faculty of Medicine, Jeddah, SAU; 4 Internal Medicine, King Abdulaziz University Faculty of Medicine, Jeddah, SAU; 5 Faculty of Medicine, King Abdulaziz University, Jeddah, SAU; 6 Section of Neurology, Department of Pediatrics, King Abdulaziz University Hospital, Jeddah, SAU; 7 Pediatrics, Taif University, Jeddah, SAU

**Keywords:** pediatric sleep questionnaire, children’s sleep habits questionnaire, sleep disorder, pediatric inpatient, sleep study

## Abstract

Background

It is possible to define sleep disorders as any disturbance in sleep timing, quality, or quantity that results in daytime distress and impairment in functioning that, in turn, affects the baseline functional status of an individual. Our study aimed to describe how sleep disorders might affect pediatric inpatients at King Abdulaziz University Hospital (KAUH) as well as estimate their prevalence (2021-2022). We assessed the sleep habits using questionnaires and analyzed and combined these data to create rankings to compare the different issues affecting sleep habits in pediatric patients.

Methodology

Two scoring systems were used in this study, namely (a) the Children’s Sleep Habits Questionnaire (CSHQ) and (b) the Pediatric Sleep Questionnaire. Analyses of the data were conducted using SPSS version 23 (IBM Corp., Armonk, NY, USA) and GraphPad Prism version 8 (GraphPad Software, Inc., San Diego, CA, USA).

Results

The prevalence of sleep disorders and their correlations were evaluated among 98 pediatric inpatients at KAUH, Saudi Arabia, between 2021 and 2022. The average duration of hospital stay was 11.97 ± 11.0 days (N = 78), and the average number of previous admissions was 2.85 ± 3.7 (N = 93).

Conclusions

According to the *sleep behavior* domain of the CSHQ, most children woke up sweating, screaming, and inconsolable during the night. Furthermore, bedtime resistance and sleep anxiety were the most prevalent sleep disturbances observed in the study population.

## Introduction

Sleep disorders are defined as any disturbance in the sleep timing, quality, or quantity that affects the baseline functional status of an individual [1,2]. According to the third edition of the International Classification of Sleep Disorders (ICSD-3), there are seven major categories of sleep disorders, namely, sleep-related breathing disorders, insomnia, parasomnia, central disorders of hypersomnolence, circadian rhythm sleep-wake disorders, sleep-related movement disorders, and other sleep disorders [3]. Although epidemiological studies have shown variations in the prevalence of pediatric sleep disorders, sleep problems are prevalent in 50% of children [4-6].

The essentiality of sleep in maintaining body health is well known and studied, especially in the pediatric population, as their physical, psychological, and mental functions are still developing [7,8] Chronic childhood sleep deprivation is considered a risk factor for impaired mental health, cognition, emotional regulation, immunity, and the development of chronic diseases in adulthood [8-11].

Decreased sleep during the hospitalization period is associated with abnormal vital signs such as blood pressure and blood glucose, in addition to increased recovery time [12-14]. Pediatric inpatients are vulnerable to poor sleep quality and less sleep than recommended during the hospitalization period [15].

According to a study conducted in Chicago, several factors in hospital environments disturb sleep in pediatric patients. These include medical interventions such as vital sign monitors, continuous staff assessment, medication administration, and noise during the night [16]. Similarly, a study conducted in Southampton, UK, on pediatric inpatients and their co-sleeping parents to objectively measure their sleep quality in the hospital and compare it with their sleep at home, found that both participants experienced a decrease in sleep quality in the hospital. Moreover, residents in the pediatric ward were exposed to a higher noise level than recommended by the World Health Organization [17]. A cohort study conducted on pediatric inpatients in Mexico found that hospitalization improved sleep in patients with previous sleep problems (PSPs). However, the hospital environment caused sleep disturbances in patients without PSPs [18].

A study in Egypt concluded that sleep disturbance, excessive daytime sleepiness, and restless leg syndrome are prevalent in pediatric patients with chronic kidney disease. However, this study did not investigate these findings in pediatric inpatients [19]. Nevertheless, there are no data on the screening prevalence of sleep disorders in pediatric inpatients and the effect of these disorders on the recovery duration and length of hospital stay [20,21].

Although many studies have investigated sleep quality in hospitals and published recommendations to control the environmental factors that decrease sleep quality among pediatric inpatients, there is an overlap between sleep disorders and sleep problems, and the published recommendations are for improving sleep quality overall, but not specifically for inpatients with sleep disorders [8,9,16,22].

As there are no previous studies in this field that have focused on the effects and prevalence of sleep disorders, specifically in pediatric inpatients, this study aimed to provide new data to pediatric, psychiatric, and sleep medicine to help develop recommendations that can improve patient care and well-being.

Our study aimed to describe how sleep disorders might affect pediatric inpatients at King Abdulaziz University Hospital (KAUH) as well as estimate their prevalence (2021-2022).

## Materials and methods

In this cross-sectional study, identical inclusion/exclusion criteria were used in two scoring systems, namely, (A) the Children’s Sleep Habits Questionnaire (CSHQ). The CSHQ has been utilized in many studies to investigate sleep habits in young children as it is a retrospective questionnaire composed of 45 elements distributed among eight domains [23]. For the purpose of the study, an abbreviated form of the CSHQ was used. It included 22 elements distributed among four major domains (bedtime, sleep behavior, waking during the night, and morning wake-up) [23]. (B) The Pediatric Sleep Questionnaire (PSQ). The PSQ contains 22 items categorized into three symptoms (hyperactive or inattentive behavior, snoring, and excessive daytime sleepiness) [24].

The PSQ and CSHQ questionnaires were translated into the Arabic language and then given to the caregivers of 98 pediatric male and female patients aged 4-14 years to be completed. The translation of the questionnaire to the Arabic language was validated by previously published studies [21].

We excluded patients who were admitted to the hospital due to sleep disorders and non-Arabic-speaking patients and caregivers.

Our study was conducted in the Department of Medicine, Psychiatry Division in KAUH, Saudi Arabia, between 2021 and 2022. Ethical approval was obtained from the Research Ethics Committee, Unit of Biomedical Ethics, King Abdulaziz University, Jeddah, and was approved by the Institutional Review Board of King Abdulaziz University (reference number: 419-21).

Statistical analysis

Data obtained in this study were analyzed using SPSS version 23 (IBM Corp., Armonk, NY, USA) and GraphPad Prism version 8 (GraphPad Software, Inc., San Diego, CA, USA).

Simple descriptive statistics were used to define the sociodemographic characteristics through counts and percentages for the categorical variables, while continuous variables were presented as means and standard deviations.

Reliability analysis was then used with a model of alpha (Cronbach) to study the properties of the measurement scales, the items that comprise the scales, and the average interitem correlation.

The chi-squared test was used to establish the relationships between categorical variables. Variables represented as means were correlated using Pearson’s correlation coefficient. Independent t-tests and one-way analysis of variance tests were employed to compare the means of two or more groups. The variables correlated were CSQH scores, PSQ scores, and bedtime and wake-up time routines of the children. These tests were performed under the assumption of a normal distribution. A p-value of <0.05 was the criterion used to discard the null hypothesis.

## Results

**Figure 1:**
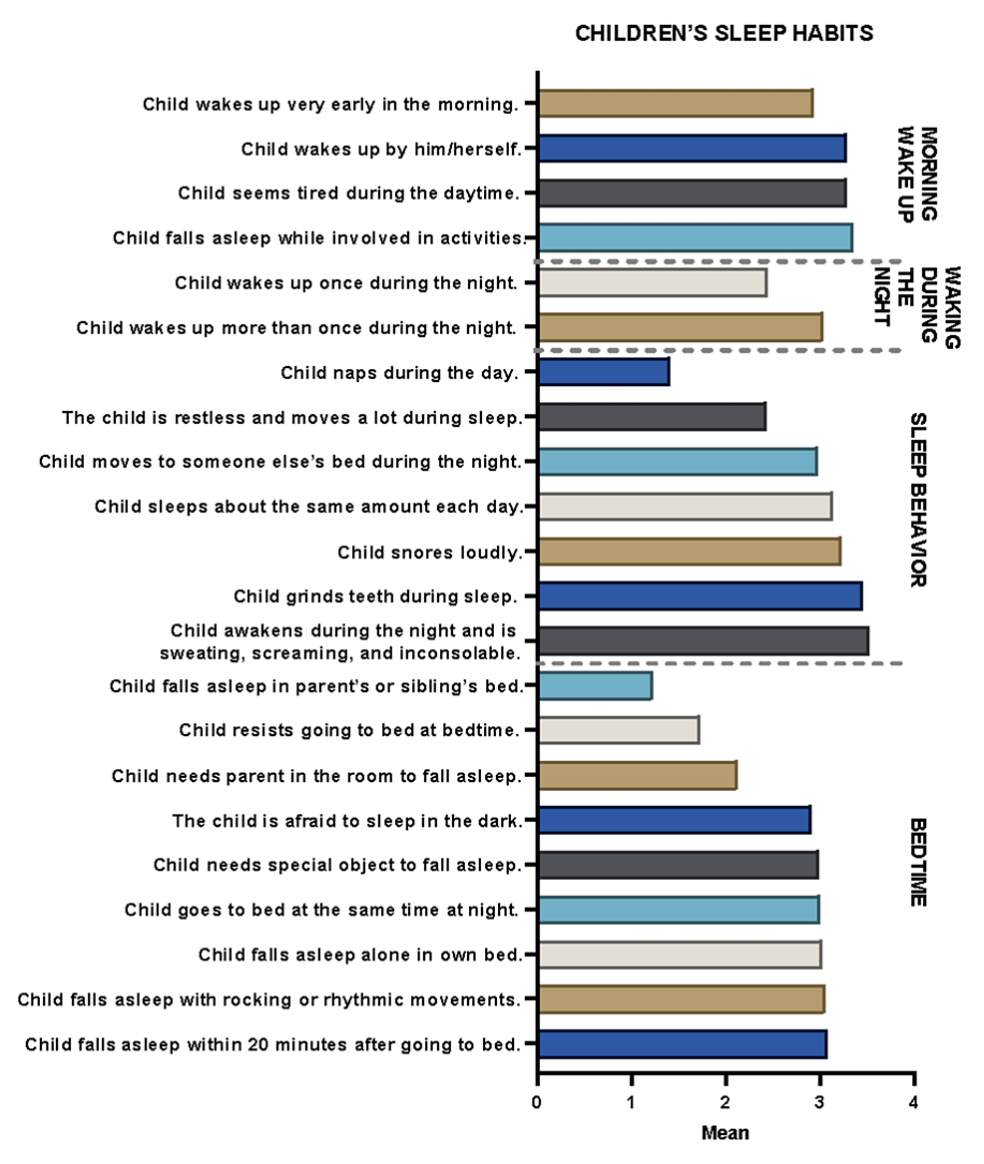
Distribution of mean scores per domain of the Children’s Sleep Habits Questionnaire of the studied patients (N = 98).

The prevalence of sleep disorders and their correlations were evaluated among 98 pediatric inpatients at KAUH, Saudi Arabia, between 2021 and 2022. Of these, 51 (53.7%) were males, and 44 (46.3%) were females. Their age ranges were toddlers (2-5 years), school-aged (6-12 years), and adolescents (13-18 years), with percentages of 30.5, 58.9, and 10.5%, respectively. Of these, 58.2% were Saudi, and 41.8% were non-Saudi. The average duration of hospital stay was 11.97 ± 11.0 days (N = 78), and the average number of previous admissions was 2.85 ± 3.7 times (N = 93).The average scores obtained by the patients for each item within each domain of the CSHQ are shown in Figure 1. The highest mean score of 3.53 ± 0.9 (N = 95) was observed for the *child awakens during the night and is sweating, screaming, and inconsolable* item under the *sleep behavior* domain, while the lowest score of 1.23 ± 1.6 (N = 95) was observed in the *child falls asleep in parent’s or sibling’s bed* item in the *bedtime* domain. In addition, reliability statistics were calculated for each domain of the CSHQ.

The frequency of answers to each item of the PSQ was evaluated (Table 1). The results showed that most patients did not respond favorably to each item. The mean scores obtained by patients for each item of the PSQ were also measured. The highest mean score of 0.37 ± 0.5 (N = 95) was observed for the *Child often interrupts or intrudes on others* item while the lowest score of 0.03 ± 0.2 (N = 95) was observed for the *Seeing the child stop breathing during the night* item.

**Table 1:** Frequency of answers to each item of the Pediatric Sleep Questionnaire of the studied patients (N = 98). Note: Counts and percentages were adjusted due to missing data.

Pediatric Sleep Questionnaire (N = 98)		Yes	No	Don’t know
While sleeping, does your child	Snore more than half the time?	19 (20.0)	73 (76.8)	3 (3.2)
	Always snore?	13 (13.7)	81 (85.3)	1 (1.1)
	Snore loudly?	8 (8.4)	85 (89.5)	2 (2.1)
	Have “heavy” or loud breathing?	16 (16.8)	77 (81.1)	2 (2.1)
	Have trouble breathing, or struggle to breathe?	15 (15.8)	79 (83.2)	1 (1.1)
Have you ever seen your child stop breathing during the night?		3 (3.2)	91 (95.8)	1 (1.1)
Does your child	Tend to breathe through the mouth during the day?	16 (16.8)	68 (71.6)	11 (11.6)
	Have a dry mouth when waking up in the morning?	22 (23.2)	62 (65.3)	11 (11.6)
	Occasionally wet in the bed?	21 (22.1)	73 (76.8)	1 (1.1)
Does your child:	Wake up feeling unrefreshed in the morning?	17 (17.9)	77 (81.1)	1 (1.1)
	Have a problem with sleepiness during the day?	17 (17.9)	77 (81.1)	1 (1.1)
Has a teacher or other supervisor commented that your child appears sleepy during the day?		10 (10.5)	79 (83.2)	6 (6.3)
Is it hard to wake your child up in the morning?		16 (16.8)	79 (83.2)	0 (0.0)
Does your child wake up with headaches in the morning?		10 (10.5)	82 (86.3)	3 (3.2)
Did your child stop growing at a normal rate at any time since birth?		24 (25.3)	64 (67.4)	7 (7.4)
Is your child overweight?		14 (14.7)	81 (85.3)	0 (0.0)
This child often	Doesn’t seem to listen when spoken to directly	24 (25.3)	68 (71.6)	3 (3.2)
	Has difficulty organizing tasks and activities	19 (20.0)	66 (69.5)	10 (10.5)
	Is easily distracted by extraneous stimuli	29 (30.5)	62 (65.3)	4 (4.2)
	Fidgets with hands or feet, or squirms in seat	31 (32.6)	57 (60.0)	7 (7.4)
	Is “on the go” or often acts as if “driven by a motor”	22 (23.2)	73 (76.8)	0 (0.0)
	Interrupts or intrudes on others (e.g., butts into conversations or games)	35 (36.8)	58 (61.1)	2 (2.1)

The association between the mean PSQ results and the sociodemographic characteristics of the patients was then determined (Table 2). The results revealed no significant association (p > 0.05) between the PSQ results of *having no sleep problems* or *having sleep problems* (breathing disorders) and age, gender, and nationality. Furthermore, the association between the mean PSQ results and CSHQ domains was measured (Table 3). The mean PSQ results were significantly associated with the *sleep behavior* domain of the CSHQ, as well as *waking during the night* and *morning wake-up* domains, with p-values of <0.001, 0.017, and 0.043, respectively.

**Table 2:** Association between the Pediatric Sleep Questionnaire and sociodemographic characteristics of the studied patients (N = 95).

Demographics		Total	Pediatric Sleep Questionnaire		P-value
			Without sleep problems	With sleep problems (breathing disorder)	
Length of hospital stay (Days)		75	11.38 ± 11.2	16.56 ± 10.7	0.197
Number of previous admissions		91	2.94 ± 3.8	2.36 ± 3.0	0.635
Age (years)	2–5	28	27 (96.4%)	1 (3.6%)	0.218
	6–12	54	45 (83.3%)	9 (16.7%)	
	13–18	10	9 (90.0%)	1 (10.0%)	
Gender	Male	50	43 (86.0%)	7 (14.0%)	0.510
	Female	42	38 (90.5%)	4 (9.5%)	
Nationality	Saudi	50	44 (88.0%)	6 (12.0%)	0.829
	Non-Saudi	38	34 (89.5%)	4 (10.5%)	

**Table 3:** Association between the Pediatric Sleep Questionnaire and Children’s Sleep Habits Questionnaire domains among the studied patients. ^a^: significant using the independent t-test at the <0.05 level.

Variables	Total	Pediatric Sleep Questionnaire		P-value
		Without sleep problems	With sleep problems (breathing disorder)	
Bedtime	95	23.30 ± 4.7	22.00 ± 4.8	0.371
Sleep behavior	95	20.92 ± 2.9	15.08 ± 4.4	<0.001^a^
Waking during the night	95	5.70 ± 2.3	3.92 ± 2.8	0.017^a^
Morning wake-up	95	13.06 ± 2.7	11.33 ± 3.0	0.043^a^

## Discussion

To our knowledge, no study has reported the prevalence of sleep disorders among pediatric inpatients children in Saudi Arabia. Therefore, this study was designed to estimate the prevalence of sleep disorders among pediatric inpatients at KAUH.

Regarding the research objective, we determined that most children woke up sweating, screaming, and inconsolable during the night according to the *sleep behavior* domain of the CSHQ, contrary to the results of Robyn et al., who found no association between CSHQ scores and risk of awakening in hospitals [10]. While the least mentioned sleep habit among pediatric inpatients was *falling asleep in their parent’s or sibling’s bed*.

Furthermore, we found that *Bedtime resistance* and *Sleep anxiety* were the most prevalent sleep disturbances when comparing our results with those of Liu et al., who used the CSHQ to examine Chinese kindergarteners’ sleep patterns and disorders [25]. Additionally, van Litsenburg et al. examined the sleep habits and problems of healthy Dutch children. They found that sleep onset delay was more common in older than in younger children, children while bedtime resistance was more commonly observed in younger children and was more common than in older children [26].

Consequently, inpatients might have more difficulty maintaining their sleep cycle than the general pediatric population experiencing difficulty falling asleep. This finding is consistent with that of Linder and Christian who investigated the effects of the hospital care environment on the sleep quality of pediatric cancer inpatients [22]. It found that sleep was significantly affected by the disruption of the sleep cycle during the night, for example, by frequent awakening and excessive noise.

Based on the PSQ results of this study, 12 patients reported more than eight positive responses, suggesting sleep problems related to breathing disorders. Despite this, the PSQ item of *seeing the child stop breathing during the night* was associated with the least prevalent sleep problems among pediatric inpatients. These results differ from a study conducted in Saudi Arabia in 2019, which investigated the prevalence of sleep-disordered breathing in primary school children using the PSQ. The study showed that the overall risk of sleep-disordered breathing was 21% [27]. The variation between our results and this study might be attributed to the pediatric populations studied, as we investigated sleep problems faced by pediatric inpatients rather than those of the general pediatric population.

Interestingly, the PSQ results were found to be significantly associated with the CSHQ domains, including *sleep behavior*, *waking during the night* (p = 0.017), and *morning wake-up* (p = 0.043) according to the independent t-test at p < 0.05 level. One implication is the possibility of benefiting from integrating both scales to develop an all-inclusive scale.

This study has a few limitations that need to be acknowledged. First, the sample size is small. We excluded non-Arabic-speaking parents, although 85% of the admissions were Arabic speaking. Second, this study was limited to inpatients at KAUH rather than multicenter patients, which limits its generalizability. Another limitation of this study is the use of a cross-sectional design. Objective measurements of sleep disturbances, such as polysomnography, may be more accurate than subjective measurements. Using questionnaires such as the PSQ and CSHQ is another limitation, as they do not replace the clinical diagnosis of an experienced physician.

The ages of the participants varied between two and 18 years, with younger children who sleep with their parents having their parents observing them during sleep. For older children who usually sleep alone, parents may be less aware of their sleeping habits.

In future investigations, we recommend comparing the sleeping habits of inpatient and outpatient children to determine whether they are affected by their surrounding environment, as well as taking into account the admitting diagnosis of the inpatients.

## Conclusions

This study estimated the prevalence of sleep disorders among pediatric inpatients using two scoring systems, namely, the CSHQ and the PSQ at KAUH, Jeddah, Saudi Arabia. In conclusion, according to the *sleep behavior* domain of the CSHQ, the majority of children woke up sweating, screaming, and inconsolable during the night. Furthermore, *bedtime resistance* and *sleep anxiety* were the most prevalent sleep disturbances among the chosen population.

Finally, the results revealed that age, sex, and nationality did not significantly influence the PSQ scores for *having no sleep problems* or *having a sleep problem* (breathing disorder). Other PSQ results, such as *sleep behavior*, *waking during the night*, and *morning wake-up*, were significantly associated with CSHQ domains.
